# Premature consent and patient duties

**DOI:** 10.1007/s11019-021-10024-5

**Published:** 2021-05-12

**Authors:** Andrew P. Rebera, Dimitris Dimitriou

**Affiliations:** AND Consulting Group, Pl. M. Broodthaers 8, 1060 Bruxelles, Belgium

**Keywords:** Informed consent, Consent, Shared decision-making, Patient duties, Medical integrity, Competence

## Abstract

This paper addresses the problem of ‘premature consent’. The term ‘premature consent’ (introduced in a 2018 paper by J.K. Davis) denotes patient decisions that are: (i) formulated prior to discussion with the appropriate healthcare professional (HCP); (ii) based on information from unreliable sources (e.g. parts of the internet); and (iii) resolutely maintained despite the HCP having provided alternative reliable information. HCPs are not obliged to respect premature consent patients’ demands for unindicated treatments. But why? What is it that premature consent patients do or get wrong? Davis has argued that premature consent patients are incompetent and misinformed. We argue that this view is not sustainable. A more plausible position asserts that premature consent threatens the integrity of the medical profession. We argue that this gives rise to a negative patient duty (to not obstruct HCPs in upholding the integrity of the medical profession) which premature consent patients fail to honour. We argue for a further positive duty of good faith engagement in shared decision-making. This implies willingness to potentially revise or justify one’s evaluative bases (core assumptions, beliefs, values, etc.). Fundamentally, the problem with premature consent patients is that certain of their evaluative bases are not open to revision. They therefore fail in their duty to participate faithfully in the shared decision-making process.

## Introduction

Informed consent requires that patients and research participants are adequately informed about proposed interventions. Healthcare professionals (HCPs) are responsible for disclosing relevant information, confirming it has been understood, and correcting misunderstandings or ignorance.^1^ However information is available from other sources. The internet provides valuable health information, but also misinformation, ill-informed opinion, and otherwise unreliable claims.^2^ How should HCPs respond to patient decisions based upon information from dubious sources?

Davis (2018) introduces the term ‘premature consent’ to denote patient decisions that are: (i) formulated prior to discussion with the appropriate HCP; (ii) based on information from an unreliable source; and (iii) resolutely maintained despite the HCP providing alternative reliable information. While there is consensus that HCPs are not obliged to respect patient demands for unindicated treatments (Brock and Wartman 1990; Pellegrino 1994; Buetow 1998; Draper and Sorell 2002; English 2005; Graber 2005; Brazier 2006; Evans 2007), there can be significant pressure do so. Respect for patient autonomy might seem to support giving way (all else equal, shouldn’t competent patients’ decisions command such respect? (Davis 2018, p. 258)); resisting demands may provoke anger and exact a psychological toll on HCPs (Nilsen and Malterud 2017); and moreover, while HCPs retain control over the treatments offered to patients, how this offering will be assessed in case of legal dispute is unclear (Cave 2020). We accept the consensus view. But what justifies refusal? What is it that premature consent patients do or get wrong? This is our central question.

In the section titled “Health information seeking, competence, and understanding” we argue against Davis’s (2018) contention that premature consent patients’ decisions do not (fully) command respect because such patients are incompetent and misinformed. A more compelling position holds premature consent wrong insofar as it undermines the integrity of the medical profession (Brazier 2006). In clarifying this position (section: “Autonomy and the integrity of the medical profession”) we argue that premature consent not only jeopardises the societal benefits secured by medical integrity, but violates a negative patient duty to not obstruct HCPs in upholding the integrity of the medical profession. In the section titled “Patient duties” we argue that premature consent patients fail also in a positive duty of good faith engagement in a process of shared decision-making.

## Health information seeking, competence, and understanding

Online health information seeking is not inherently problematic: patients ought to inform themselves (Gauthier 2005); and policymakers encourage ‘eHealth literacy’ (Norman and Skinner 2006; Paige et al. 2017) and the use of reliable online resources (eHealth Task Force 2012).^3^ Perhaps the problem lies with how premature consent patients find and evaluate online health information?

Accessing reliable online health information is less simple than it seems. People struggle to distinguish reliable from unreliable sources (Convie et al. 2020, p. 11). Longstanding concerns about the gatekeeping role of search engines touch on results bias or manipulation and a lack of transparency (Introna and Nissenbaum 2000; Granka 2010). Search and browsing histories, social networking profiles, and location may all influence search results (Holone 2016; Loh 2016; Chang et al. 2018). Privacy-aware users have sought to avoid filter bubbles, profiling, micro-targeting, and tracking by using privacy-friendly search engines. But this may provoke other issues: findings from Ghezzi et al. (2019) suggest that privacy-focused search engines may return results of lower health information quality. These difficulties affect everyone to some extent. So there is no reason to suppose premature consent patients disproportionately subject to them.

### Are premature consent patients incompetent?

Competence assessments are inherently normative and concern both *content* (in that one may believe irresponsibly) and *process* (in that one’s manipulation of beliefs may be unjustified) (Banner 2012). Four capacities are taken to jointly indicate competence: (i) communicating a choice; (ii) understanding relevant information; (iii) appreciating the situation and its likely consequences; and (iv) manipulating information rationally (Appelbaum and Grisso 1988). While (i) and (iii) are not particularly relevant to premature consent, (ii) and (iv) capture the importance of content and process.

Davis incorporates (ii) and (iv) into the concept of *deliberation*: good deliberation requires careful consideration of one’s options, based on evaluation of relevant information, its credibility, and the trustworthiness of its sources (Davis 2018, p. 259). Premature consent patients do not deliberate well because they misevaluate the credibility of their information and the trustworthiness of its sources. But this is not a failing of either content or process alone.^4^ They are deficient in getting and maintaining their beliefs in good order (process) *because* they are deficient in evaluating medical information and its sources (content). This, Davis claims, is a kind of incompetence.

For Davis, premature consent patients seriously misevaluate the relative reliability of internet- and HCP-sourced information. But it cannot *always* be an error (or at least not a *gross* one) to overestimate the reliability of an internet source. In various scenarios, a patient could reasonably favour an online source: they may not consider the HCP expert in the relevant field; advice from another HCP may corroborate the online source; or the online source may be independently credible (e.g. health authority-approved). In such cases, the patient can have ‘recognisable reasons’ for a decision, i.e. the kind that can be intelligible to a third party (Banner 2013). These can be indicative of competence if they fit into a coherent account of the agent’s overall behaviour. In premature consent cases, the patient has received information from an *unreliable* source. But this does not preclude their having recognisable reasons for favouring that information. The premature consent patient may, for example, be competent in identifying reliable sources, but mistaken on a given occasion (which, on that occasion, partially explains their stubborn resistance to the HCP’s recommendations). They will, on such occasions, have recognisable reasons for their position. If the patient has recognisable reasons, they are, plausibly, competent. If they do not have recognisable reasons, they may well be incompetent. But to reason without recognisable reasons is indicative of incompetence quite independently of any peculiarity of premature consent. Thus Davis’s position on incompetence fits only *some* premature consent cases: those in which we anyway have independent reasons to doubt the patient’s competence.

In cases where the patient has recognisable reasons for their position, it is not their procedural reasoning that is at fault. The problem is that they have a false belief. They possibly believe false claims from an unreliable source^5^; and they *do* harbour false beliefs about the reliability of the source. Perhaps, then, they are misinformed.

### Are premature consent patients adequately informed?

Being adequately informed of relevant information is a requirement for valid informed consent. Davis (2018, pp. 262–3) gives two arguments for the claim that premature consent patients are not adequately informed.

The first relies on a distinction between ‘being’ and ‘having been’ informed of relevant information. A subject *has been informed* of some relevant information *p* if *p* has been disclosed to them and they understand it; they *are informed* of *p* if, additionally, they *believe* it (Davis 2018, p. 262). According to Davis (2018, p. 262), “You need to *be* informed in order for your consent to command respect, and that’s what’s missing in cases of premature consent” (because the patient does not believe the information disclosed by the HCP).

However, as Davis (2018, p. 262) himself notes, an HCP’s duty of disclosure is discharged when the patient *understands* the information (see e.g. The World Medical Association 2013): belief is not required. Perhaps, then, Davis has specific beliefs in mind. A subset of propositions disclosed in informed consent are *so* pertinent that a patient’s failure to believe them *is* problematic (e.g. concerning risks in a clinical trial, or where a patient is delusional^6^). For these, HCPs are obliged to “disabuse an individual of even a resolutely held false belief” (Faden et al. 1986, p. 310). Other propositions are so well established that contrary beliefs are demonstrably false (Faden et al. 1986, p. 253). Davis’s position is plausible for propositions of these two kinds. But if the truth of such propositions is easily demonstrable, the problem of premature consent shouldn’t arise in relation to them (the patients can be readily disabused of these false beliefs; or if they are unresponsive to the demonstrations, their overall competence is in question independently of questions of premature consent). Difficult premature consent cases will then concern false beliefs about ‘inherently contestable’ matters, where there is room for reasonable disagreement (Faden et al. 1986, p. 310ff). But it is implausible that false beliefs about inherently contestable matters undermine the capacity to consent.^7^

According to Davis’s second argument, patients ought to know “who (or what) are the right *sources* of information” (Davis 2018, p. 263), whereas premature consent patients apparently do not. This, however, collapses into positions already addressed. Call the supposedly missing knowledge about reliable sources ‘*p*’. Since HCPs must disclose all relevant information, if *p* is relevant (e.g. it is a list of reliable information sources), the HCP should disclose it (Convie et al. 2020, p. 11). If *p* is disclosed, cases in which the patient fails to believe it are addressed above in this subsection. If *p* is not such that the HCP is obliged to disclose it—e.g. it concerns general principles of source-evaluation—then the patient’s failure to believe it calls into question their overall competence *independently* of any issues specific to premature consent.

This renders the account less than satisfactory. A satisfactory account of what the premature consent patient does or gets wrong should attribute to them some error, deficiency, or similar (call it *e*), such that: (1) the patient’s being subject to *e* explains their tendency to premature consent in particular (as opposed to some other failing); (2) if the patient were *not* subject to *e*, they would not be especially prone to premature consent. For Davis, *e* is a deficiency in the understanding or application of general principles of source-evaluation. This satisfies condition 1: the patient is at increased risk of forming false beliefs because they cannot identify *unreliable* sources; and they are at increased risk of not revising their false beliefs because they cannot identify *reliable* sources by which to make the revision. They are, thus, prone to premature consent in particular. But the account does not satisfy condition 2: a patient could be competent at identifying reliable sources of information (i.e. not subject to *e*) and yet still be prone to premature consent. This is because one can be competent in identifying reliable sources without being infallible (as was noted in the previous subsection) and, moreover, one might simply be very stubborn, arrogant, or reluctant to reconsider one’s views. This is a deficiency, but it is not incompetence, and it has nothing to do with one’s facility in identifying reliable sources of information.

## Autonomy and the integrity of the medical profession

Informed consent is typically cashed out in terms of *disclosure*, *understanding*, *capacity/competence*, *voluntariness*, and *consent* (Beauchamp and Childress 2019, p. 119) (some accounts omit the fifth). These promote autonomy in decision-making which protects against unwanted interventions and paternalism (though see O’Neill 2003). Effective informed consent processes empower patients by requiring that they provide an ‘active authorisation’ (Faden et al. 1986, p. 277ff) of an intervention. In actively endorsing an intervention, a patient assumes their share of responsibility for its consequences (because autonomy and responsibility go hand in hand). This holds equally for HCPs. In deploying their professional expertise (e.g. in defining treatment options), an HCP becomes a “de facto moral accomplice in what happens to her patient” (Pellegrino 1994, p. 52). This brings professional autonomy into view.

Pellegrino (1994, pp. 51–2) identifies three senses of HCP autonomy:

(1) autonomy as a person, which gives moral status to the physician’s [HCP’s] personal moral values and conscience; (2) autonomy as a physician, which gives moral status to the physician’s knowledge and obligation to use it wisely and well; and (3) autonomy as a member of a profession, of a moral community with collective obligations to patients and society.^8^

By insisting upon a decision the HCP advises against, the premature consent patient potentially undermines HCP autonomy in sense 1. But it is implausible that every premature consent case poses a serious question of conscience for the HCP. For most cases, the threat is more plausibly to HCP autonomy in sense 2. But senses 2 and 3 are intertwined. Suppose a patient’s insistence threatens an HCP’s autonomy in sense 2 by discounting their professional expertise and reducing them to a “mere service provider” (Brazier 2006, pp. 416, 420). HCP roles are incompatible with ‘mere’ service provision. But explaining why takes us from HCP autonomy in sense 2 to sense 3: premature consent patients threaten the autonomy of HCPs as members of the medical profession.

### The integrity of the medical profession

Professions involve specialised knowledge or skills; their members require specific education, training, and possibly certification or licensing; professions often define the limits and nature of their own work; they can be self-regulating and are frequently bound to particular ethical codes (Hoogland and Jochemsen 2000, pp. 458–9). Professions enjoy the privileges they do (e.g. self-regulation) because of their vital societal role: HCPs, in exercising their professional knowledge, promote health and wellbeing (Hoogland and Jochemsen 2000, p. 459). On this way of looking at it, professional autonomy is the ‘price’ of delivering these health and wellbeing benefits. But is the price fair? Is medical integrity genuinely a condition on delivery of the benefits?

Brazier (2006) argues emphatically for the incompatibility of ‘mere service provision’ and medical integrity. A window cleaner (a service provider) is under no obligation to take the job of cleaning your windows, nor even to explain their refusal. If HCPs were mere service providers, they too could select among cases, refusing those deemed difficult, mundane, or whatever, turning away patients considered awkward, unable to pay, and so forth (Brazier 2006, p. 420). A patient could struggle, in such a scenario, to find a willing HCP. But in the real world, while an HCP may refuse to perform an intervention in specific circumstances (e.g. for reasons of conscience), overall the medical profession functions as a ‘provider of last resort’: its collective ethical code ensures that everyone gets treated, no matter who they are or what their circumstances. Thus, as a mere service provider, an HCP could refuse any patient for any reason; but as a medical professional they cannot refuse for *just any* reason, but only very serious reasons (such as those of conscience). On entering the profession, HCPs become what Draper and Sorell (2002) call ‘captive helpers’: they are obliged to treat the patient in front of them,^9^ unless for some valid matter of conscience.^10^ It is this *captivity* that distinguishes the medical profession from mere service providers (Brazier 2006, p. 421).

### Captive helpers

If captivity distinguishes the medical profession from mere service providers, and if premature consent threatens to reduce the former to the latter, then captivity is fundamental to the explanation of what premature consent patients do or get wrong. But what is the precise connection?

Draper and Sorell (2002) argue that patients are obliged to “do what they can to limit the captivity of doctors” (2002, p. 349) and to act so that “doctors are *willing* rather than captive helpers” (2002, p. 350). The argument calls on an analogy between friendship and the HCP-patient relationship (Draper and Sorell 2002, pp. 348–50). Friends are obliged to help one another even when it is inconvenient. This is, say Draper and Sorrell, a variety of captivity. But the captivity of friendship has limits: “The devotion of friends and relations can be strained to breaking point, and the people who make use of it are under some obligation to try and limit their calls on it” (2002, p. 349). So just as friends have an obligation to minimise the burdens of friendship-captivity, so patients have an obligation to minimise the burdens of HCP-captivity: specifically, to “follow reasonable medical advice” and “enable doctors whose sound advice is ignored to withdraw from [the relationship]” (2002, p. 350). Premature consent patients fail in at least one of these obligations. In so doing they make HCP-captivity worse. This is what they get wrong.

However, the analogy between friendship and the HCP-patient relationship is loose at best. First, where the medical profession collectively ensures that no patient is abandoned, friendships offer no such guarantee (there is no ‘friend of last resort’). Second, friendship requires equality of the parties (a friendship vitiated by condescension, hero-worship, or other asymmetries is, to that extent, not genuine), but HCP-patient relationships are inherently unequal (the HCP is an expert, the patient normally not; the patient seeks counsel, the HCP offers it; the HCP occupies a position of power, the patient a position of vulnerability). It is risky to draw conclusions from a loose analogy.

A further complication attends the claim that captivity obliges friends to minimise avoidably burdensome behaviour. One should, obviously, recognise that by being continually burdensome in avoidable ways one will likely lose friends and alienate people. But, in this case, fear of the consequences no more grounds the obligation to minimise the behaviour that might bring them about than the desire to avoid punishment grounds the obligation to behave lawfully. One should minimise avoidably burdening one’s friends *out of respect for them*, not as a means of keeping them. Analogously, patients should cooperate with HCPs simply out of respect for them. But this obligation is independent of captivity (for notice that one should cooperate with one’s *non-captive* window-cleaner out of respect for them too). So Draper and Sorrell’s argument that certain patient duties “flow from benefitting from a captive relationship” (2002, pp. 349–50) is unconvincing.

Captivity imposes duties upon HCPs, but it is unclear how it could directly impose duties upon patients.^11^ Perhaps its role is simply to highlight how medical integrity establishes a kind of contract between the medical profession and society (Brazier 2006) aimed at securing valuable health benefits. Thus if premature consent challenges medical integrity, it undermines the contract and jeopardises the benefits thus secured. Yet premature consent would be objectionable even if it didn’t undermine the societal contract or have undesirable societal consequences.^12^ So the appeal to the social contract is only an *incomplete* account of the wrongness of premature consent: it captures the harm to society (jeopardised societal benefits); it captures the harm to the profession (undermining its integrity and instigating a kind of breach of contract); but it fails to capture the harm to the HCP. But what harm?

HCPs incur duties upon joining their profession, including those entailed by captive helper status. They are, in general, duty-bound to uphold the integrity of the medical profession (by treating all patients equally, with honesty and respect, in confidentiality, in light of best-practice and the evidence-base, with due regard for public health and the allocation of scarce resources, and so on). This being so, premature consent patients may be cast as seeking, for no valid reason, to have HCPs ignore or subvert their duty to uphold the integrity of their profession. HCPs have a *pro tanto* obligation to meet this duty and so, equally, everyone else has a *pro tanto* obligation to not obstruct them. Therefore: (1) the premature consent patient has a negative duty to not obstruct the HCP in carrying out their duty of medical integrity; (2) insofar as they unjustifiably insist on a decision against the best advice of the HCP, the premature consent patient fails to honour this negative duty; (3) other things being equal, it is wrong to fail to honour one’s duties; so (4) other things being equal, the premature consent patient acts wrongly. (And they act wrongly even if there is no potential or actual risk to the societal benefits that medical integrity secures.)

## Patient duties

The integrity of the medical profession imposes duties on HCPs. These imply a negative duty on everyone else not to be obstructive. Premature consent is wrong insofar as it involves failing to meet this negative duty. But this is not all that is wrong. Patients have *positive* duties too: a propensity to premature consent is, we argue, a propensity to fail in respect of positive duties concerning *shared decision-making*.

Patient duties range from being truthful with HCPs, to respecting public health advice, to reporting unethical conduct by HCPs (see e.g. American Medical Association (AMA) (2019), opinion E-1.1.4). They flow from a number of sources (English 2005), including the nature of the HCP-patient relationship. Meyer (1992, p. 545) characterises the HCP-patient relationship as a “cooperative partnership”, the “very essence” of which is “commitment to the practice of bilateral decision-making”. A division of labour is implied: broadly, HCPs propose possible treatments and patients select among them. But an HCP cannot make the optimal delineation of treatment options without input from the patient; and the patient cannot make an informed selection without input from the HCP. In this information exchange, there is a potential disconnect between the technocratic, rationalised knowledge of the medical profession and the narrative, meaning- and value-infused knowledge of patients (Hoogland and Jochemsen 2000, pp. 461–2). HCPs must guard against supposing that their expertise affords the only or best perspective on patients’ best interests. But patients too must guard against a consumerist stance according to which HCPs and the healthcare system exist solely to satisfy their preferences (Buetow 1998, p. 245). Between these excesses is a space in which shared decision-making can exist.

### Shared decision-making

Shared decision-making is set out in detail by Sandman and Munthe (2010). It is distinctive in extending HCP-patient collaboration beyond discussion of patient options and preferences right through to the decision itself (Sandman and Munthe 2010, p. 61) (a more fitting moniker might be ‘shared decision-*taking*’). Yet shared decision-making is not a ‘hard and fast’ model with fixed criteria: it is better understood as an ideal to which decision-making models approximate (Sandman and Munthe 2010, p. 78). Decision-making models that most faithfully exemplify shared decision-making emphasise ‘deliberation’—which is a joint attempt to arrive at a well-founded decision—leading to consensus (Sandman and Munthe 2010, p. 74). Deliberation essentially involves *high-level dynamics*, namely “discussion where arguments and reasons have to be presented, compared and evaluated”, in which the parties’ evaluative bases are genuinely open to revision (Sandman and Munthe 2010, p. 73).^13^

Shared decision-making is not without critics. An important objection concerns its connection with informed consent. Shared decision-making inevitably draws comparison with informed consent because a significant number of healthcare decisions involve an autonomous authorisation of some kind. But Beauchamp and Childress (2019), to take a notable example, argue that such decisions simply cannot be shared:

Approval and authorization belong to the patient, not to a physician or research investigator, even when extensive shared dialogue has occurred. […] Approving and authorizing are not shared in an appropriate model of informed consent […]. (Beauchamp and Childress 2019, pp. 119–120)

Although approval and authorisation do belong to the patient alone, this shows only that the shared decision-making and informed consent processes are not identical. It does not preclude that shared decision-making models certain stages of the informed consent process (namely those dedicated to decision-making rather than authorising). *Making* a decision and *authorising* it are not the same. The HCP and patient could arrive at a jointly endorsed position via a shared decision-making process, with the patient then independently and unilaterally providing the authorisation necessary for informed consent. On this view, to *endorse* a decision is to *recommend* it or *recognise it as optimal*, and to *authorise* a decision to *give permission that it be acted upon*. Beauchamp and Childress’s point is respected because while HCPs can, in these terms, endorse a decision, they cannot authorise it (though they may be able to veto it).

If the collaborative character of the HCP-patient relationship imposes communicative responsibilities on each party, and if shared decision-making, which essentially involves high-level dynamics, is the best way of meeting those responsibilities, then HCPs and patients have duties of good faith engagement in shared decision-making: of collaboration in joint reasoning where both parties are in principle willing to revise their evaluative bases. Premature consent patients fail to meet this duty of good faith engagement.^14^ Specifically, in their stubborn unresponsiveness to HCPs’ reasoned advice, they demonstrate an unwillingness to engage in high-level dynamics: their evaluative bases are closed to revision.

Two cases should be distinguished, however. Where a patient is *unwilling* to engage in good faith high-level dynamics, they fail in their duty. But there may also be cases in which a patient is not unwilling to engage—indeed they genuinely wish to be cooperative—but is nonetheless closed-minded or subject to some cognitive fault that obstructs the process of shared decision-making.^15^ In this case, the patient *is* engaging in good faith, but the engagement misfires, and a shared decision cannot be arrived at. However, in these circumstances, we no longer have a case of premature consent. Premature consent involves a decision taken prior to receiving an HCP’s advice and maintained despite it. But here, with the assumption that the patient has engaged in good faith, their initial (premature) decision has been effectively set aside: in undertaking to engage cooperatively in the shared decision-making process, they undertook to arrive at a *fresh* decision (it may turn out to be the same one) *with* the HCP. So this is not a case of a premature decision stubbornly maintained, but of good faith disagreement. It is a *hard case* no doubt, but not a case of premature consent.^16^

### Non-engagement and wrong-engagement

In going through an informed consent process, patients incur a duty to engage in shared decision-making. But duties may be felt as burdens. In a sense, the price of healthcare is the burden imposed by the duty to engage. This, one might suggest, is unfair.

In providing informed consent, one inevitably has to engage, in some manner and to some extent, with an HCP. As an unavoidable condition of informed consent, this engagement is not analogous to a price or fee: fees can be waived, but there is no waiving informed consent, and so no waiving the engagement.^17^ If there is a burden, it is unavoidable. If there is unfairness, it is balanced out by the benefits of informed consent.

But a second objection follows. Granted informed consent requires engagement of some kind; but shared decision-making imposes a particular burden through the requirement to engage in high-level dynamics (which is potentially a difficult process). What if a patient fails to meet their duty to engage in the high-level dynamics of shared decision-making not, as in premature consent, through a ‘static’ unwillingness to re-evaluate their position, but through a disinclination to engage in that way (e.g. a preference for acquiescing to whatever the HCP suggests)? Here, the duty of engagement is apparently not met, yet intuitively the patient is not acting wrongly (or at least not egregiously).

Patients and HCPs share responsibility for the decisions they collectively make. A patient’s duty to collaborate via shared decision-making is a duty to support the HCP in their endeavour (which is also the patient’s) to arrive at an appropriate decision. But this duty can be met in more than one way. What we might call ‘full engagement’—i.e. complete buy-in to the process (providing all information, being fully transparent, actively and openly deliberating and reconsidering evaluative bases, etc.)—offers the most support to the HCP; less full forms of engagement offer less support. But the duty is only to support the other party: the extent of the support is not fixed. A patient who is disinclined to give full engagement does not automatically fail in the duty. At a minimum, if they engage sufficiently that the interaction results in valid informed consent, the duty of engagement has been met: the parties have arrived at a position both are willing to endorse and which the patient is willing to authorise.^18^

One can fail in one’s duty to engage in two ways. ‘Non-engagement’ involves utter uncooperativeness, acquiescence, or passivity to the point of absence. Informed consent is not possible under these conditions. A non-engaging patient will likely be judged incompetent. ‘Wrong-engagement’ involves an element of dysfunction in the collaboration (e.g. persistently misfiring communication, untruthfulness, inconstancy, etc.). Premature consent is a variety of wrong-engagement (a refusal to re-evaluate or justify one’s position).

## Conclusion

What is wrong with premature consent? What is it that premature consent patients do or get wrong?

We argued against Davis (2018), who claims that premature consent patients are neither fully competent nor adequately informed. We then reconstructed a position according to which premature consent threatens the integrity of the medical profession. For Brazier (2006), the integrity of the medical profession is incompatible with ‘mere’ service provision: HCPs, unlike service providers, are ‘captive helpers’ (Draper and Sorell 2002). However, we argued, while captivity imposes duties upon HCPs, it is unclear how it could directly impose duties upon patients. Rather, we appealed to a negative patient duty (to not obstruct HCPs in upholding the integrity of the medical profession) which premature consent patients fail to honour.

Finally, we argued that patients have a positive duty of good faith engagement in shared decision-making. This implies a willingness to collaborate in decision-making with the HCP, and to potentially revise or justify one’s evaluative bases (core assumptions, beliefs, values, etc.). Fundamentally, the problem with premature consent patients is that certain of their evaluative bases—notably their core assumptions as to the relative reliability of key sources of information—are not open to revision. Through this ‘wrong-engagement’ they fail in their duty to participate faithfully in the shared decision-making process.

## Data Availability

Not applicable.

## Footnotes

1 Henceforth ‘patients’ refers to both patients and clinical research participants; ‘HCPs’ refers to both HCPs and clinical researchers.

2 Widespread unreliable health information is not new with the internet (compare direct-to-consumer advertising in healthcare, e.g. Murray et al. (2003)). But the internet is a (if not *the*) primary health information source for many people nowadays, so ‘Dr Google’ is our focus.

3 Some HCPs find internet-informed patients threatening (Kim and Kim 2009), but online health information seeking is frequently well-motivated (Attfield et al. 2006; Bowes et al. 2012) and constructive engagement with internet-informed patients can strengthen HCP-patient relationships (Iverson et al. 2008; Hou and Shim 2010; Tan and Goonawardene 2017).

4 Not solely of content, for they do not necessarily believe falsely; but not solely of process, for the steps of their reasoning may be coherent.

5 They *may* have formed false beliefs based on the unreliable source, but this is not essential for premature consent (they could have only true beliefs and a premature consent case *still* arise).

6 Banner (2012, p. 1041) cites *Norfolk & Norwich Healthcare (NHS) Trust v W (1996)*, in which a patient refused an emergency Caesarean section because she denied she was pregnant.

7 Banner (2013, 72–3) cites UK case law showing that, in UK law at least, decisions can even be based on *irrational* — let alone merely *false* — beliefs without thereby indicating incapacity, so long as no mental impairment can be shown.

8 See also The World Medical Association (2018).

9 Captivity implies that the HCP must treat the patient (other things equal): it doesn’t imply anything about what treatment they should deliver. There is no implied obligation to do whatever the patient requests.

10 Hence Draper and Sorell (2002, p. 347): “The captivity of doctors works on two levels: on the one hand, individual doctors are only exceptionally allowed to break off a relationship with a patient; on the other hand, even if a particular doctor-patient relationship proves impossible or is brought to an end, the profession as a whole will continue to provide care for the patient”.

11 Might captivity directly impose upon patients a duty not to exploit someone else’s captivity? Possibly. But such a duty is subsumed under the more general duty to cooperate with HCPs out of respect for them—a duty which, as argued above, would apply even if they were not captive (compare window-cleaners).

12 In at least some cases, premature consent surely either doesn’t jeopardise societal benefits or does so only to a negligible extent. Of course, undermining the contract between the medical profession and society could also be considered wrong for non-consequentialist reasons.

13 Deliberation is contrasted with ‘negotiation’, which is characterised as “static” because “no party has any necessary incentive to reconsider his or her evaluative basis (ideals, preferences, values, etc.)” (Sandman and Munthe 2010, p. 73). Note that this concept of *deliberation* is not intended to bear any relation to Davis’s (2018, p. 259).

14 To fail to engage in good faith shared decision making is not a form of incompetence (competence does not require engagement in shared decision-making). Hence there is no overlap between ours and Davis’s positions on this point.

15 We thank an anonymous reviewer for pressing this case upon us.

16 What should be done in such cases? If additional time, advice from trusted third parties, or similar steps do not bring about a shared decision, it may be necessary to refer the patient to a different HCP. In theory, an extremely resolute patient could be passed from HCP to HCP to HCP … and so on: at some point the courts may have to decide (though this is obviously very much the extreme and one supposes that after a second or third opinion, most patients would accept that they cannot have the treatment they desire).

17 Informed consent can be waived in certain circumstances, but this is irrelevant for present purposes.

18 It is possible that less than full patient-engagement results in less than optimal treatment. So we must concede to the objection that, in order to access optimal healthcare, one has to ‘play the game’. But this is only to say that to access a thing one must meet the conditions attendant upon accessing it. If there is unfairness in *this*, it is minimal.
